# Benzothiazole DNA gyrase inhibitors and their conjugates with siderophore mimics: design, synthesis and evaluation†

**DOI:** 10.1039/d3ra08337c

**Published:** 2024-01-18

**Authors:** Martina Durcik, Cristina D. Cruz, Mariano Andrea Scorciapino, Janez Ilaš, Päivi Tammela, Matteo Ceccarelli, Lucija Peterlin Mašič, Tihomir Tomašič

**Affiliations:** a University of Ljubljana, Faculty of Pharmacy Aškerčeva cesta 7 1000 Ljubljana Slovenia tihomir.tomasic@ffa.uni-lj.si; b Drug Research Program, Division of Pharmaceutical Biosciences, Faculty of Pharmacy, University of Helsinki P. O. Box 56 (Viikinkaari 5 E) FI-00014 Helsinki Finland; c Department of Chemical and Geological Sciences, University of Cagliari, Cittadella Universitaria di Monserrato – S. P. 8 km 0.700 09042 – Monserrato (CA) Italy; d Department of Physics and IOM/CNR, Sezione di Cagliari, University of Cagliari, Cittadella Universitaria di Monserrato – S. P. 8 km 0700 09042 – Monserrato (CA) Italy

## Abstract

Benzothiazole-based bacterial DNA gyrase and topoisomerase IV inhibitors are promising new antibacterial agents with potent activity against Gram-positive and Gram-negative bacterial strains. The aim of this study was to improve the uptake of these inhibitors into the cytoplasm of Gram-negative bacteria by conjugating them to the small siderophore mimics. The best conjugate 18b displayed potent *Escherichia coli* DNA gyrase and topoisomerase IV inhibition. The interaction analysis of molecular dynamics simulation trajectory showed the important contribution of the siderophore mimic moiety to binding affinity. By NMR spectroscopy, we demonstrated that the hydroxypyridinone moiety alone was responsible for the chelation of iron(iii). Moreover, 18b showed an enhancement of antibacterial activity against *E. coli* JW5503 in an iron-depleted medium, clearly indicating an increased uptake of 18b in this bacterial strain.

## Abstract 2

Potent DNA gyrase and topoisomerase IV inhibitor bearing a siderophore mimic moiety displays improved antibacterial activity against *Escherichia coli* under iron-depleted conditions.

## Graphical abstract

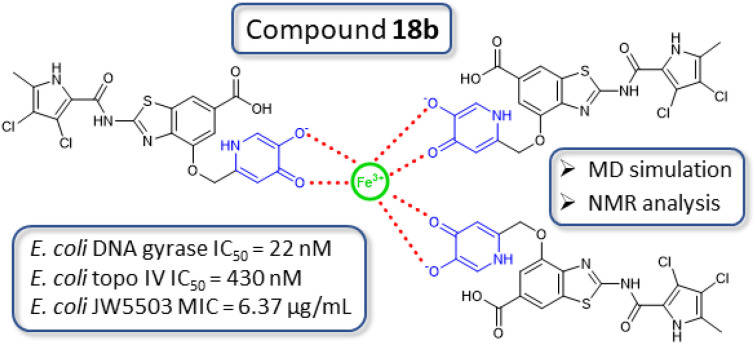

## Introduction

Antimicrobial resistance (AMR) is one of the greatest challenges of modern medicine, and the number of deaths attributable to AMR is steadily increasing.^1^ Due to the continued emergence of resistant bacteria, particularly in the ESKAPE (*Enterococcus faecium*, *Staphylococcus aureus*, *Klebsiella pneumoniae*, *Acinetobacter baumannii*, *Pseudomonas aeruginosa*, *and Enterobacter species*) group of pathogens, the development of novel therapeutic agents that act *via* novel mechanisms of action is of utmost importance.^2^ DNA gyrase and topoisomerase IV are validated targets for antibacterial drug discovery. These bacterial type IIA topoisomerases are responsible for maintaining the correct topology of the DNA molecule during its replication, transcription, decatenation, and repair.^3,4^ The DNA gyrase consists of two catalytic GyrA subunits and two GyrB subunits with ATPase activity, whereas the topoisomerase IV consists of two catalytic ParC subunits and two ATP-binding ParE subunits. Both enzymes exert their enzymatic activity as functional heterotetramers in the form A_2_B_2_.^3,5^ The clinically used fluoroquinolones^6^ and spiropyrimidinetriones (*e.g.* zoliflodacin),^7^ which are in clinical development, bind in complexes bordered by DNA, GyrA (ParC) and GyrB (ParE), whereas gepotidacin, a member of the NBTI class,^8^ binds to DNA and GyrA (ParC). The GyrB and ParE subunits, which are critical for providing energy for enzymatic reactions through ATP hydrolysis, are inhibited by the clinically used novobiocin, which has been withdrawn from therapy due to side effects and development of resistance.^9^ Fobrepodacin (SPR720) and DS-2969b are GyrB/ParE inhibitors currently in clinical development for the treatment of nontuberculous mycobacterial infections^10^ and against *Clostridium difficile* infections,^11^ respectively.

Recently, we developed the benzothiazole class of ATP-competitive dual DNA gyrase and topoisomerase IV inhibitors that exhibit potent antibacterial activity against Gram-positive (G+) and Gram-negative (G−) problematic ESKAPE pathogens.^12–15^ These resistant G+ and G− bacteria are known to be responsible for the majority of nosocomial infections. Because they are highly resistant to clinically available antibiotics,^16,17^ most of these bacterial strains are also on the priority lists of WHO and CDC.^18,19^ The problem is challenging especially for G− species, which are protected by the additional outer membrane (OM), a real physical barrier for molecules to reach the periplasm.^20^ Although porins expressed in the OM constitute the main path for molecules to penetrate, their permeability might not be sufficient to have a minimal internal accumulation.^21^ Thus other strategies are required, such as the exploitation of active siderophore transporters.^22^

We have previously also designed and evaluated conjugates of the (*S*)-4,5,6,7-tetrahydrobenzothiazole-2,6-diamine-based GyrB/ParE inhibitors^23,24^ with small siderophore mimics^25^ as a strategy to improve low cell-wall penetration of G− bacteria.^26–28^ Siderophores, which are usually catechol or hydroxamic acid derivatives, are secondary microbial metabolites synthesized to sequester iron(iii).^29^ Bacteria synthesize siderophores and release them into the host environment to take up very limited amounts of freely available iron during infection.^30^ Subsequently, the siderophore in complex with iron(iii) is transported through the bacterial cell wall by iron uptake proteins. Conjugation of the antibacterial agent with the siderophore or siderophore mimic (*e.g.*, catechol, hydroxypyranone, hydroxypyridone or dihydroxypyridone) can be used to hijack the bacterial iron-uptake machinery to increase the periplasmic or cytoplasmic concentration of the antibiotic and thus, enhance its antibacterial activity.^22,31–33^ This concept was also clinically validated, when in November 2019 the FDA approved cefiderocol, a siderophore cephalosporin antibiotic, for the treatment of complicated urinary tract infections. Cefiderocol is also being studied in phase 3 clinical trials for the treatment of nosocomial pneumonia and infections caused by carbapenem-resistant G− bacteria.^34,35^

In the present work, we conjugated our potent, dual-targeting benzothiazole-based GyrB/ParE inhibitors with small siderophore mimics and investigated their DNA gyrase and topoisomerase IV inhibition, antibacterial activity, and binding of iron(iii) by NMR spectroscopy. In addition, the binding mode of the most potent conjugate was evaluated by molecular dynamics simulation and analysis of the interactions in the ATP-binding site of *E. coli* GyrB.

## Results and discussion

### Design and synthesis

**Fig. 1:**
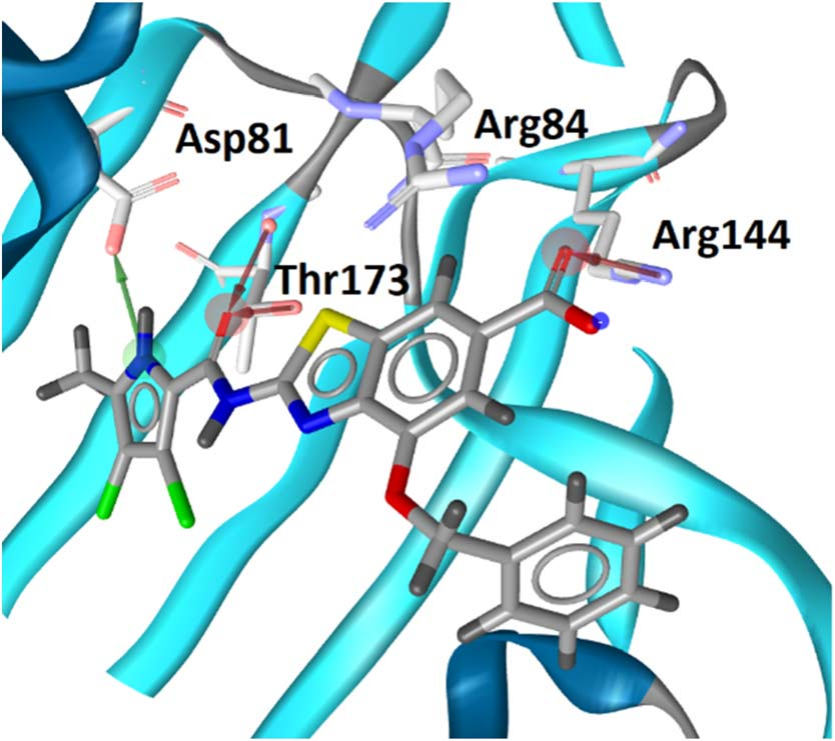
The binding mode of inhibitor B (in grey sticks) in complex with *Staphylococcus aureus* GyrB24 (in blue cartoon; PDB entry: 6TCK). For clarity, only amino acid residues forming hydrogen bonds or cation–π interaction are shown as grey sticks. Water molecule is presented as a red sphere. Hydrogen bonds are shown as green (hydrogen bond donor) and red (hydrogen bond acceptor) arrows.

The design of new DNA gyrase inhibitor–siderophore mimic conjugates started from our potent GyrB/ParE inhibitors A and B (Table 1) that exhibited antibacterial activity against G+ and G− ESKAPE pathogens, limited potential for resistance development and efficacy in *in vivo* infection models.^13,14,36,37^ The co-crystal structures of benzothiazole GyrB inhibitors in complex with *S. aureus* GyrB showed that the pyrrolamide moiety forms two hydrogen bonds with Asp81 (*S. aureus* GyrB numbering) and crystal water molecule as well as an extensive network of hydrophobic contacts. The benzothiazole core is engaged in the formation of cation–π interaction with Arg84 and substitution at position 4 allows for improving the physico-chemical properties and binding affinity by formation of additional interactions in the lipophilic floor of the binding site. Moreover, the carboxylate at position 6 forms a strong salt bridge with Arg144 (Fig. 1).^13,14,36,37^

**Table 1:** *Escherichia coli* DNA gyrase and topoisomerase IV inhibition by compounds 10, 12, 18a, and 18b

ID	Structure	IC_50_ [nM]	
		*E. coli* DNA gyrase	*E. coli* topoisomerase IV
A	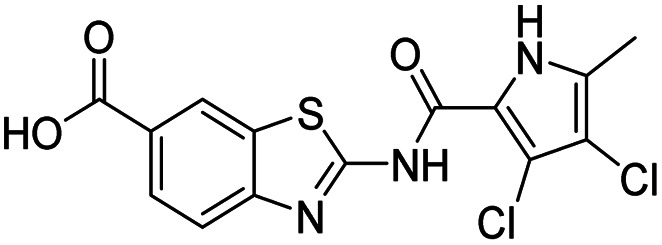	13 ± 0	500 ± 280
B	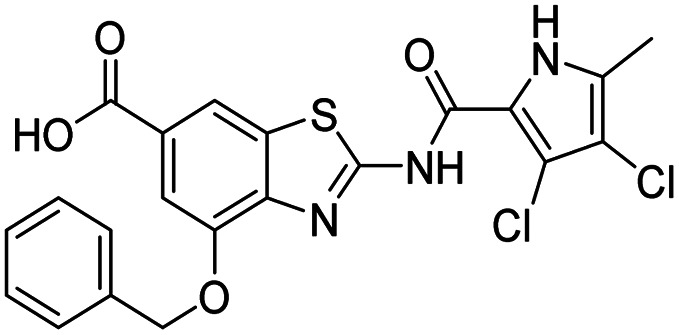	<10	350 ± 50
10	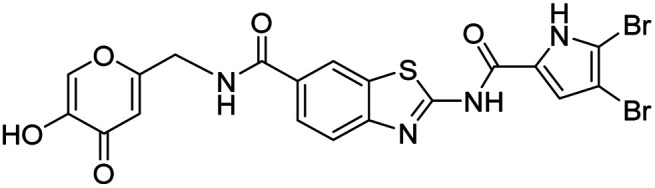	1420 ± 180	>10 000
12	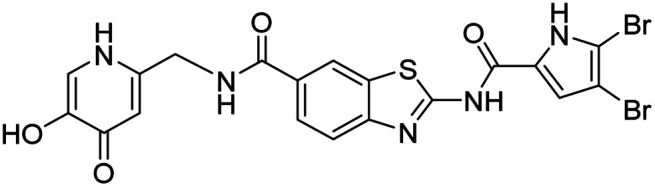	1700 ± 360	>10 000
18a	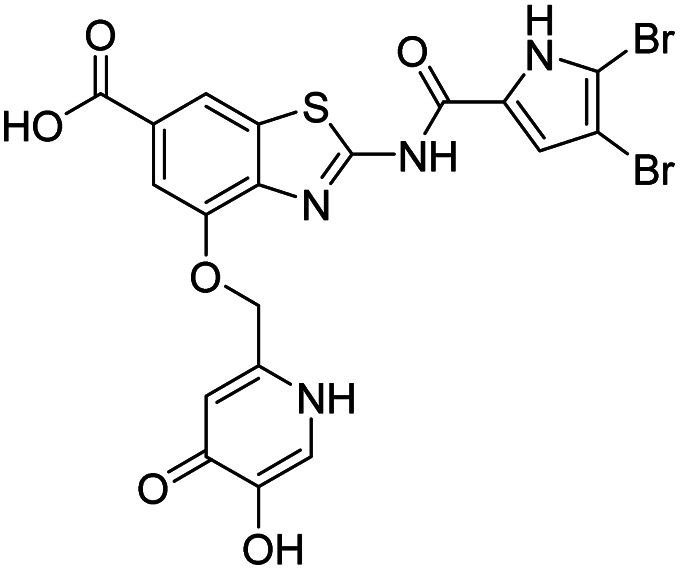	12 ± 5	1280 ± 820
18b	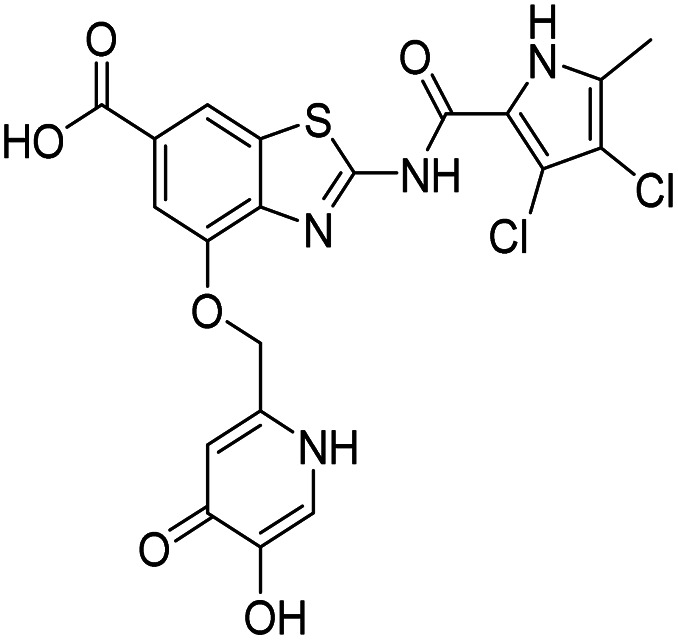	22 ± 6	430 ± 20

In the design of conjugates with siderophore mimics, we considered two different approaches. In the first design strategy, the 6-carboxylic acid was reacted with the 2-(aminomethyl)-5-hydroxy-4*H*-pyran-4-one or 2-(aminomethyl)-5-hydroxypyridin-4(1*H*)-one to form the amide derivatives 10 and 12. In the second strategy, a siderophore mimic was incorporated into position 4 of the benzothiazole core to obtain compounds 18a and 18b. In both approaches we anticipated that the siderophore mimic would form attractive interactions within the GyrB/ParE ATP-binding site and therefore, its cleavage from the inhibitor would not be a prerequisite for enzyme inhibition, as previously reported for some GyrA inhibitors conjugated to siderophores.^38–40^ We used 4,5-dibromo- or 3,4-dichloro-5-methylpyrrole moiety as both form similar interaction network in the GyrB binding site. The advantage of our approach is also that the resulting conjugates have molecular weight around 500 and therefore, better drug-like properties than previously reported ciprofloxacin–siderophore conjugates with cleavable and non-cleavable linkers.

**Scheme 1:**
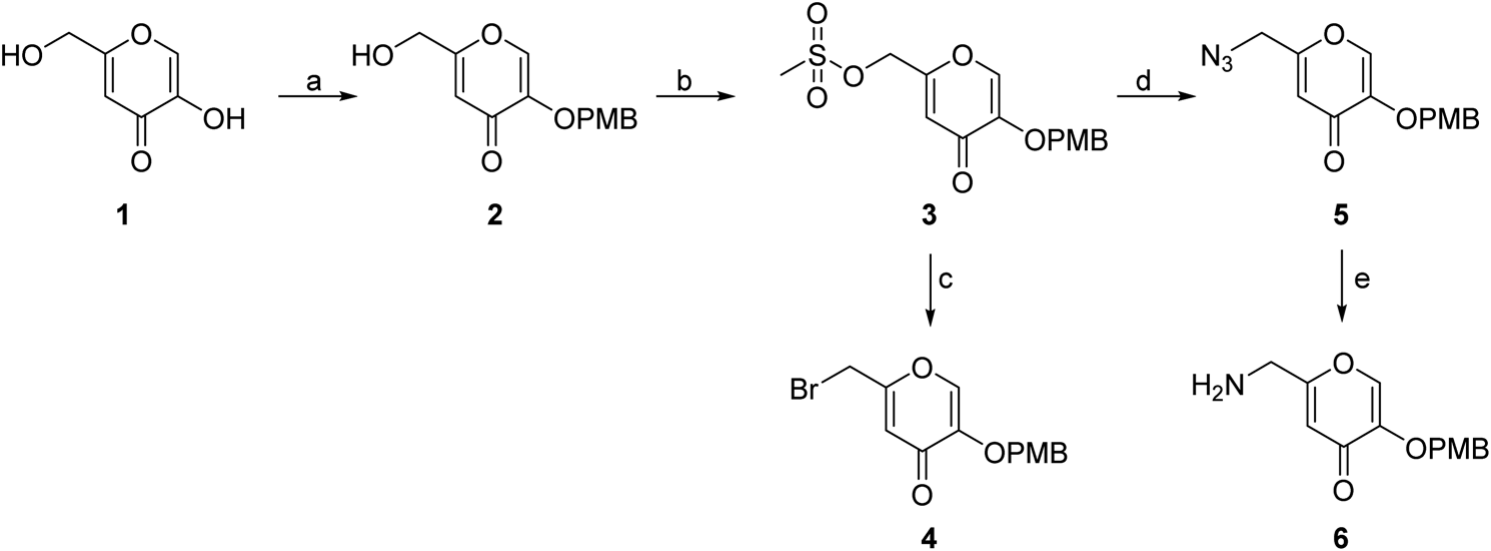
Synthesis of siderophore fragments 4 and 6. Reagents and conditions: (a) 4-methoxybenzyl chloride, K_2_CO_3_, dry DMF, 60 °C, 4 h (45.6%); (b) methanesulfonyl chloride, Et_3_N, DCM, 20 °C, 4 h; (c) NaBr, DMF, 20 °C, 16 h (69.0% over two steps); (d) NaN_3_, KI, DMF, 20 °C, 20 min (84.4%); (e) PPh_3_, MeOH, 20 °C, 1 h (37.1%). PMB – *p*-methoxybenzyl.

The synthesis of the siderophore mimic building blocks 4 and 6 started from kojic acid (1) (Scheme 1).^41^ The hydroxyl group at position 3 of 1 was selectively protected using the 4-methoxybenzyl (PMB) chloride in the presence of potassium carbonate in *N*,*N*-dimethylformamide (DMF) to obtain compound 2. Hydroxyl group of the hydroxymethylene group at position 5 of compound 2 was then activated with methanesulfonyl chloride to mesylate 3, which was converted in nucleophilic substitution with the sodium bromide to bromide 4 or with the sodium azide to azide 5. The latter was converted to amine 6 using Staudinger reaction conditions.

**Scheme 2:**
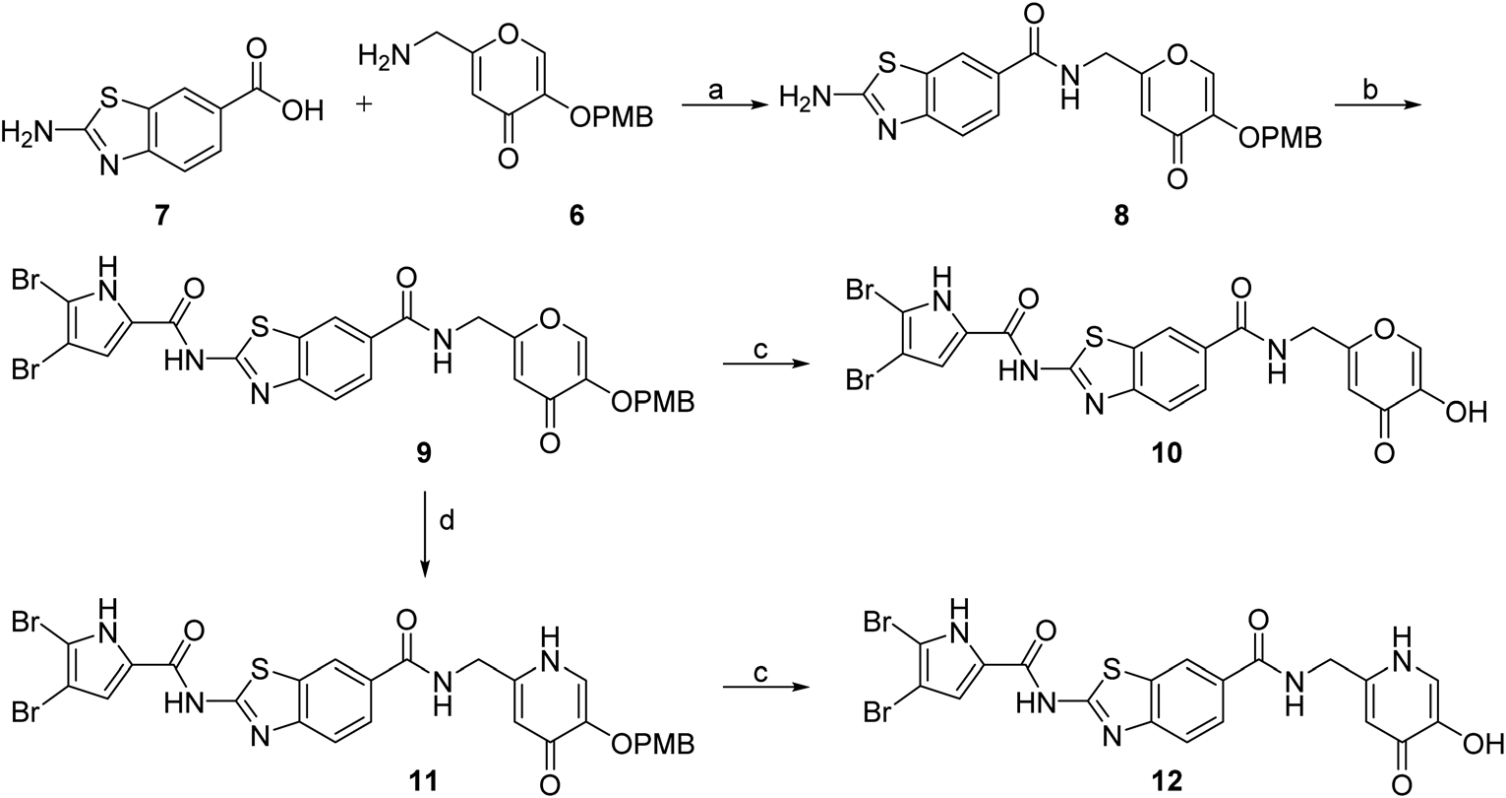
Synthesis of siderophore mimic conjugates 10 and 12. Reagents and conditions: (a) EDC, HOBt, NMM, DMF, 0 °C to 20 °C, 16 h (72.4%); (b) 2,2,2-trichloro-1-(4,5-dibromo-1*H*-pyrrol-2-yl)ethan-1-one, Na_2_CO_3_, DMF, 80 °C, 16 h (75.0%); (c) 1 M HCl in acetic acid, acetic acid, 20 °C, 16 h (84.8–88.9%); (d) NH_3_ (25% aq. solution), MeOH, 70 °C, 48 h (87.9%).

DNA gyrase inhibitor–siderophore mimic conjugates were first prepared by attaching the siderophore mimic to the carboxylic acid group at position 6 on the benzothiazole ring (Scheme 2). Firstly, EDC/HOBt-promoted amide coupling between the 2-aminobenzothiazole-6-carboxylic acid (7) and siderophore mimic building block 6 gave compound 8, which was in the next step coupled with the 2,2,2-trichloro-1-(4,5-dibromo-1*H*-pyrrol-2-yl)ethan-1-one in the presence of sodium carbonate as a base in DMF to yield pyrrolamide 9. Final conjugate 10 was prepared by the removal of the 4-methoxybenzyl protecting group of 9 using HCl in acetic acid. 4*H*-Pyrane-4-one derivative 9 was also converted to the pyridin-4(1*H*)-one derivative 11 by refluxing 9 in aqueous ammonia solution. DNA gyrase–siderophore conjugate 12 was finally prepared by deprotection of the 4-methoxybenzyl group of 11 under acidolysis conditions.

**Scheme 3:**
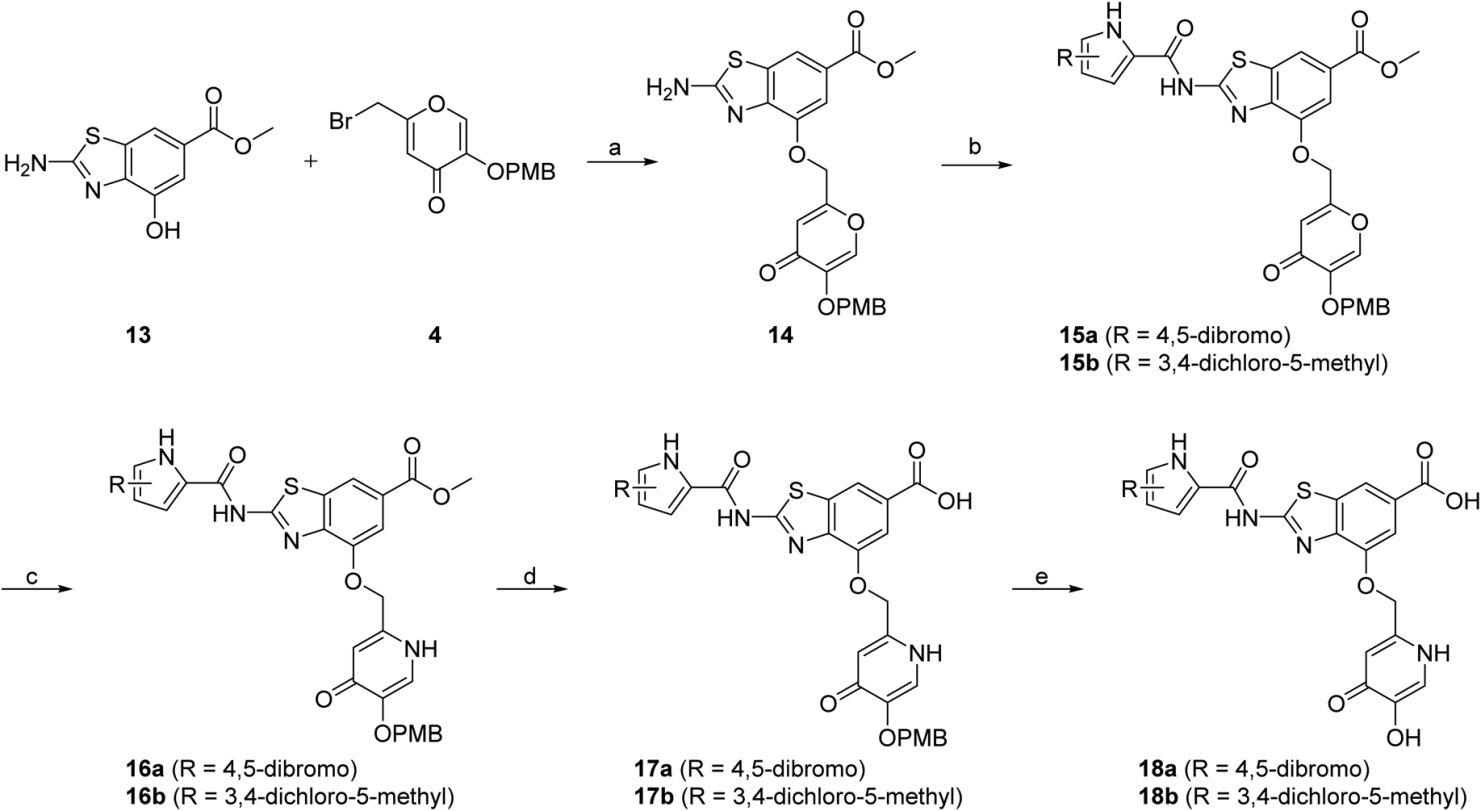
Synthesis of siderophore mimic conjugates 18a and 18b. Reagents and conditions: (a) K_2_CO_3_, CH_3_CN, 60 °C, 16 h (60.5%); (b) 2,2,2-trichloro-1-(4,5-dibromo-1*H*-pyrrol-2-yl)ethan-1-one (for 15a) or 2,2,2-trichloro-1-(3,4-dichloro-5-methyl-1*H*-pyrrol-2-yl)ethan-1-one (for 15b), Na_2_CO_3_, DMF, 80 °C, 16 h (24.5–77.8%); (c) NH_3_ (25% aq. solution), MeOH, 60 °C, 3 h, then 20 °C, 16 h (or 60 °C, 16 h – for 16b) (32.0–33.0%); (d) 1–2 M NaOH, 1,4-dioxane (for 17a) or MeOH (for 17b), 40 °C, 16 h (33.3–63.7%); (e) 1 M HCl in acetic acid, acetic acid, 20 °C, 40 h (52.4–62.3%). PMB – *p*-methoxybenzyl.

In addition to the attachment of siderophore mimics to position 6 of the benzothiazole, we also considered introducing such structural elements to position 4 of this scaffold (Scheme 3). The synthesis of final compounds 18a and 18b started from methyl 2-amino-4-hydroxybenzothiazol-6-carboxylate (13)^42^ that was alkylated with the building block 4 under basic conditions to obtain compound 14. Pyrrolamides 15a and 15b were obtained by reacting amine 14 with the 2,2,2-trichloro-1-(4,5-dibromo-1*H*-pyrrol-2-yl)ethan-1-one or 2,2,2-trichloro-1-(3,4-dichloro-5-methyl-1*H*-pyrrol-2-yl)ethan-1-one, respectively, in the presence of sodium carbonate in DMF. 4*H*-Pyrane-4-one derivatives 15a and 15b were refluxed in ammonium hydroxide to obtain pyridin-4(1*H*)-one derivatives 16a and 16b. To prepare final conjugates 18a and 18b the order of removal of protecting groups was important. First, methyl ester of 16a and 16b was cleaved using alkaline hydrolysis to get 17a and 17b, followed by acidolysis to yield final products 18a and 18b.

### *In vitro* enzyme inhibition

All final compounds were first tested for their inhibitory activity in the *E. coli* DNA gyrase supercoiling assay and the *E. coli* topoisomerase IV relaxation assay (Table 1). The enzyme inhibition data showed that the amide formation at position 6 (compounds 10 and 12) had a detrimental effect on the inhibition of *E. coli* topoisomerase IV (IC_50_ values above 10 μM) and resulted in weak inhibition of *E. coli* DNA gyrase with IC_50_ values in the low micromolar range. These results are consistent with our previous study in which bioisosteric replacements of the carboxylate group or its substitution resulted in weaker DNA gyrase and topoisomerase IV inhibition.^14^ In the same study, we also showed that substituents at position 4 of the benzothiazole scaffold in potent inhibitors can have different chemical properties. These observations were confirmed with compounds 18a and 18b, which carried the siderophore mimic at position 4. These compounds inhibited *E. coli* DNA gyrase in the low nanomolar range and displayed only slightly weaker *E. coli* topoisomerase IV inhibition than did the parent compound B (Table 1). With compounds 18a and 18b, we confirmed our hypothesis that it is possible to maintain potent enzyme inhibition after introduction of the siderophore mimic into the structure of the GyrB/ParE inhibitor.

### Molecular modeling

**Fig. 2:**
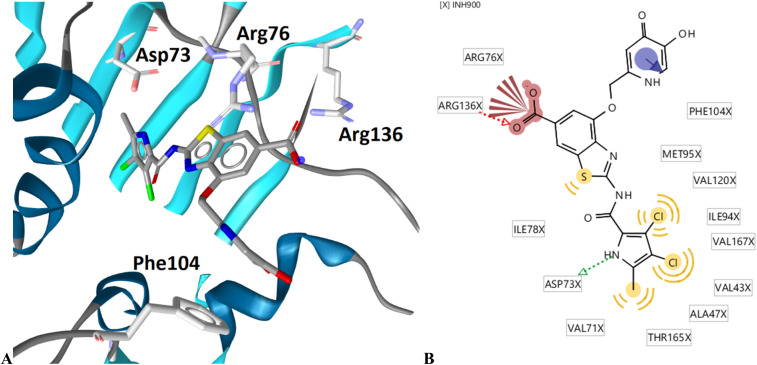
Representative snapshot from molecular dynamics trajectory. (A) Compound 18b (in grey sticks) in the binding site of *E. coli* GyrB (PDB entry: 4WUB). (B) Schematic representation of the pharmacophore model. Pharmacophore features are presented as follows: hydrophobic features as yellow spheres, hydrogen bond donor as a green arrow, hydrogen bond acceptor as a red arrow, negative ionizable as a red star and aromatic rings as a blue disc.

The binding modes of 18b in the ATP-binding sites of *E. coli* GyrB and ParE were studied by a combination of molecular docking calculations, molecular dynamics simulation and interaction analyzes. Inhibitor 18b was first docked to *E. coli* GyrB (PDB entry: 4WUB^43^) and ParE (PDB entry: 1S16 (ref. 44)) ATP-binding sites. These two co-crystal structures were used because they contain the flexible loop in the lipophilic floor of the enzymes that can interact with the substituent at position 4 of the benzothiazole core. The binding mode of 18b was consistent with the co-crystal structures of benzothiazole-based GyrB/ParE inhibitors in complex with *S. aureus* GyrB. In *E. coli* GyrB, the pyrrolamide moiety formed hydrogen bonds with Asp73 and Thr165 and several hydrophobic interactions in the hydrophobic pocket. A salt bridge was formed between the 6-carboxylate group and Arg136 and a cation-π interaction was formed between the benzothiazole ring and Arg76 guanidinium group. The docking binding mode of 18b in complex with *E. coli* GyrB was used as a starting point for the 100 ns MD simulation. The MD trajectory was then analyzed using structure-based pharmacophore modeling in LigandScout. Analysis of the MD simulation trajectory showed that the siderophore mimic bound at position 4 of the benzothiazole core is quite flexible and forms hydrogen bonds with the Ala100–Tyr109 residues of the flexible loop. It forms additional π-stacking with the Phe104 side chain (Fig. 2), which is present in 18% of the simulation time. This analysis clearly showed the important contribution of the siderophore mimic to the binding affinity.

### Antibacterial activity

We also tested the antibacterial activity of the siderophore mimic-bearing GyrB/ParE inhibitors 10, 12 and 18b against wild type G− *E. coli*, *P. aeruginosa* and *A. baumannii* in iron-depleted cation-adjusted Mueller Hinton broth (ID-CAMHB) and iron-supplemented CAMHB broth (CAMHB + Fe). Cefiderocol, a siderophore cephalosporin, was used as a positive control. Compound 18a was only tested on *E. coli* in both media conditions. Iron-depleted conditions were used to determine the effects of the attached siderophore mimics on antibacterial activities, since bacteria under low iron conditions produce and secrete siderophores to take up iron from the extracellular environment.^32^ In general, compounds 10, 12, 18a and 18b displayed rather weak antibacterial activity at 50 μM in iron-supplemented and iron-depleted media, which indicated MIC values > 50 μM (Table 2). We have previously observed that our benzothiazole GyrB/ParE inhibitors are strongly effluxed from the G− bacterial cells and that this effect is especially pronounced in *E. coli*.^14^ Therefore, compounds 10, 12, and 18b were also tested against *E. coli* JW5503, which is a *tolC* deletion mutant strain with a defective efflux pump. Compound 18b showed an MIC value of 25 μM (12.7 μg mL^−1^) under iron-supplemented conditions. Improvement of antibacterial activity of 18b against efflux-defective *E. coli* JW5503 with an MIC value of 12.5 μM (6.37 μg mL^−1^) was observed under iron-depleted conditions. For cefiderocol, which was used as a positive control, similar twofold improvement of antibacterial activity was observed in iron-depleted medium (Table 2). These results demonstrate the contribution of the siderophore mimic to cell wall permeation rendering to a more potent antibacterial activity of 18b, however, efflux remains to be a major issue of the benzothiazole GyrB/ParE inhibitors. The antibacterial activity of 18b was weaker than that of parent compounds A and B, suggesting that the incorporation of the siderophore mimic has a limited contribution. In our recent study, we also showed that benzothiazole-based GyrB inhibitors with potent activity against G− strains have a topological surface area (TPSA) of less than 120 Å^2^.^36,37^ Replacement of the phenyl ring of inhibitor B (TPSA = 100.0 Å^2^) by the oxygen- and nitrogen-rich siderophore mimic in 18b results in a significantly higher TPSA of 149.4 Å^2^, which may be one of the reasons for the weaker antibacterial activity of 18b despite the presence of the siderophore mimic in its structure.

**Table 2:** Antibacterial activity of 10, 12, 18a, and 18b against *E. coli*, *P. aeruginosa* and *A. baumannii*^a^

	*E. coli* ATCC 25922		*P. aeruginosa* ATCC 27853		*A. baumannii* ATCC 19606		*E. coli* JW5503	
	−Fe	+Fe	−Fe	+Fe	−Fe	+Fe	−Fe	+Fe
Minimum inhibitory concentrations [μM]								
10	>50	>50	>50	>50	>50	>50	>50	>50
12	>50	>50	>50	>50	>50	>50	>50	>50
18a	>50	>50	n.t	n.t	n.t	n.t	n.t	n.t
18b	>50	>50	>50	>50	>50	>50	12.5	25
Cefiderocol	0.25	0.25	0.25	1	0.25	2	0.0625	0.125

a −Fe: iron depleted cation adjusted Mueller Hinton broth media (ID-CAMHB), +Fe: cation adjusted Mueller Hinton broth media + Fe (CAMHB + Fe), nt.: not tested.

### NMR spectroscopy of 18b–iron(iii) complex

**Fig. 3:**
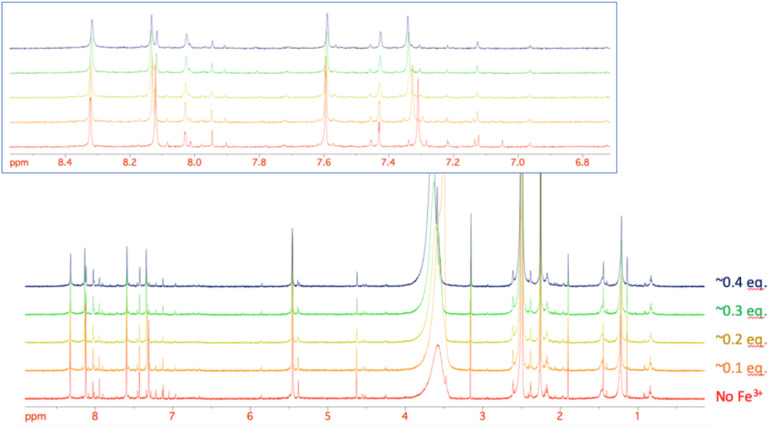
The stacking plot of NMR spectra of 18b after stepwise addition of FeCl_3_ to a DMSO solution of 18b. The inset shows the aromatic region of 18b.

**Fig. 4:**
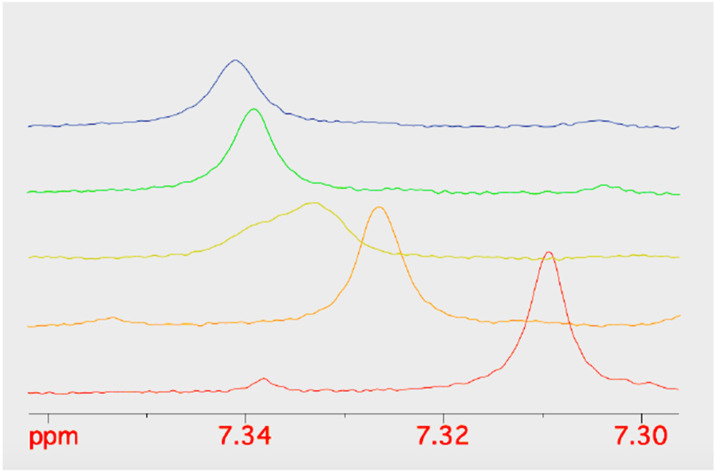
The stacking plot of NMR spectra of the pyridinone proton at 7.3 ppm of 18b after stepwise addition of FeCl_3_ to a DMSO solution of 18b.

Given the improved antibacterial activity of 18b against the *E. coli* JW5503 in iron-depleted medium, its potential for chelation of iron(iii) was studied by NMR spectroscopy. To a DMSO-*d*_6_ solution of 18b, FeCl_3_ in steps of 0.1 equivalents of Fe^3+^ was added (Fig. 3). A very small shift was observed for all the resonances in the spectrum with increasing Fe^3+^ concentration (Fig. 3), which can be attributed to the strong acidity of the ferric chloride solution. In fact, the hydroxypyridinone resonances showed the largest shift among 18b resonances. However, while all the resonances did not change their profile along this short titration (apart from the progressive broadening due to the increasing concentration of the paramagnetic Fe^3+^ ion in solution), the two hydroxypyridinone resonances showed a difference. Fig. 4 shows how the single resonance (red spectrum) progressively broadened (orange) and passed through a clear convolution of two components (yellow) at about 0.2 equivalents of Fe^3+^. Then, the signal progressively returned to a single component (green) and started to progressively broaden (blue). This is the typical trend observed when two forms are in a relatively slow exchange, with the molar fraction of the first one progressively decreasing from 1 to 0, while the other progressively and complementarily increases from 0 to 1. One of the two forms is the free ligand, which is maximum in the absence of ferric ions. With increasing the latter, the free ligand is progressively transformed to the ligand–metal complex. Our experiment showed that the signals for a free ligand disappeared by the addition of 0.4 equivalents of FeCl_3_, with the half-equivalence point around 0.2. This clearly suggests the formation of complexes with stoichiometry L_3_M (where L is the ligand and M the metal). Moreover, in the range investigated (ligand excess), all of the other resonances appeared to be insensitive to the formation of the complex, which shows that only the hydroxypyridinone moiety is exploited in the complex formation. These two observations agree with each other. Each hydroxypyridinone is expected to use two O donors to coordinate the metal ion, thus, in the complex L_3_M, the three hydroxypyridinones are enough to complete the typical octahedral configuration around Fe^3+^.

## Conclusions

We have prepared new conjugates of our antibacterially active GyrB/ParE inhibitors with 2-(aminomethyl)-5-hydroxy-4*H*-pyran-4-one and 2-(aminomethyl)-5-hydroxypyridin-4(1*H*)-one siderophore mimics. These mimics were attached to two different positions and their incorporation to the position 4 of the benzothiazole scaffold resulted in potent DNA gyrase inhibition, whereas the inhibition of topoisomerase IV was slightly weaker compared with the parent inhibitors. The most potent inhibitor 18b showed a twofold enhancement of antibacterial activity against the efflux-defective *E. coli* strain JW5503, but was only weakly active against wild-type *E. coli*, *P. aeruginosa* and *A. baumannii.* The results with wild-type and efflux-defective *E. coli* strains indicated that efflux remains a problem for benzothiazole-based GyrB/ParE inhibitors and will be the focus of future optimization.

## Experimental section

Chemicals were obtained from Acros Organics (Geel, Belgium), Sigma-Aldrich (St Louis, MO, USA), Apollo Scientific Ltd (Stockport, UK), Fluorochem Ltd (Derbyshire, UK), and Enamine Ltd (Kyiv, Ukraine) and were used without further purification. Analytical TLC was performed on silica gel Merck 60 F254 plates (0.25 mm), using visualization with UV light and spray reagents. Column chromatography was carried out on silica gel 60 (particle size, 240–400 mesh). ^1^H and ^13^C NMR spectra were recorded at 400 MHz and 101 MHz, respectively, on a Bruker AVANCE III 400 spectrometer (Bruker Corporation, Billerica, MA, USA) in DMSO-*d*_6_ or CDCl_3_ solutions, with TMS as the internal standard. Mass spectra were obtained using an ADVION expression CMS^L^ mass spectrometer (Advion Inc., Ithaca, USA) and high-resolution mass spectra were obtained using VG Analytical Autospec Q Micromass mass spectrometer (Fisons, VG Analytical, Manchester, UK) (compounds 5, 8–12) or Exactive Plus Orbitrap mass spectrometer (Thermo Fisher Scientific, Waltham, MA, USA) (compounds 18a and 18b). HPLC purity analyses were performed on a 1260 Infinity II LC system (Agilent Technologies Inc., Santa Clara, CA, USA). A Waters XBridge C18 column was used (3.5 μm, 4.6 mm × 150 mm), with flow rate of 1.5 mL min^−1^ and sample injection volume of 10 μL. The mobile phase consisted of acetonitrile (solvent A) and 0.1% formic acid in 1% acetonitrile in ultrapure water (solvent B). The gradient (defined for solvent A) was: 0–1.0 min, 25%; 1.0–6.0 min, 25–98%; 6.0–6.5 min, 98%; 6.5–7.5 min, 98–25%; 7.5–10.5 min, 25%. All active tested compounds were more than 95% pure as established by HPLC, unless indicated otherwise.

### Synthesis of compounds

#### 2-(Hydroxymethyl)-5-((4-methoxybenzyl)oxy)-4*H*-pyran-4-one (2)^25^

5-Hydroxy-2-(hydroxymethyl)-4*H*-pyran-4-one (1, 5.00 g, 35.2 mmol) and K_2_CO_3_ (10.7 g, 77.4 mmol) were suspended in DMF (65 mL), flushed with argon and heated to 60 °C. 4-Methoxybenzyl chloride (5.25 mL, 38.7 mmol) was added dropwise and the reaction mixture was stirred at 60 °C for 4 h. The reaction mixture was poured on a mixture of water and ice (1 : 1, 500 mL) and left overnight. The formed crystals were filtered off and dried. Yield: 4.21 g (45.6%); orange crystals; mp: 112 °C.

#### (5-((4-Methoxybenzyl)oxy)-4-oxo-4*H*-pyran-2-yl)methyl hydrogen sulfate (3)

To a solution of 2 (4.12 g, 15.7 mmol) in DCM (55 mL) cooled on an ice bath triethylamine (4.40 mL, 31.7 mmol) was added and the mixture was stirred for 20 min. The reaction mixture was heated to rt, methanesulfonyl chloride (1.20 mL, 15.5 mmol) was added and the reaction mixture was stirred at rt for 3 h. After the reaction was finished dichloromethane (30 mL) was added and the organic phase was washed with brine (140 mL). Organic phase was dried over Na_2_SO_4_, filtered and the solvent removed *in vacuo*. The crude oily product was used in the next step without further purification.

#### 2-(Bromomethyl)-5-((4-methoxybenzyl)oxy)-4*H*-pyran-4-one (4)

To a suspension of 3 (4.12 g, 12.04 mmol) in DMF (10 mL) NaBr (3.23 g, 31.4 mmol) was added and the reaction mixture was stirred at rt overnight. Ethyl acetate (80 mL) was added and the organic phase was washed with brine (2 × 100 mL), dried over Na_2_SO_4_, filtered and the solvent removed *in vacuo*. The crude product was purified with flash column chromatography using dichloromethane/methanol (20 : 1) as eluent. Yield: 2.70 g (69.0%); white crystals; mp: 107 °C. ^1^H NMR (400 MHz, DMSO-*d*_6_) *δ* [ppm] 3.76 (s, 3H), 4.56 (s, 2H), 4.86 (s, 2H), 6.57 (s, 1H), 6.95 (d, *J* = 8.7 Hz, 2H), 7.35 (d, *J* = 8.7 Hz, 2H), 8.26 (s, 1H).

#### 2-(Azidomethyl)-5-((4-methoxybenzyl)oxy)-4*H*-pyran-4-one (5)

To a suspension of 3 (3.29 g, 9.66 mmol) in DMF (10 mL) NaN_3_ (1.26 g, 19.3 mmol) and catalytic amount of KI were added and the reaction mixture was stirred at rt for 20 min. Ethyl acetate (80 mL) was added and the organic phase was washed with 1% citric acid (2 × 50 mL) and brine (50 mL), dried over Na_2_SO_4_, filtered and the solvent removed *in vacuo*. Yield: 2.33 g (84.4%); light brown oil; mp: 107 °C. ^1^H NMR (400 MHz, CDCl_3_) *δ* [ppm] 3.83 (s, 3H), 4.16 (s, 2H), 5.04 (s, 2H), 6.43 (s, 1H), 6.92 (d, *J* = 8.8 Hz, 2H), 7.34 (d, *J* = 8.8 Hz, 2H), 7.56 (s, 1H). HRMS (ESI^+^) *m*/*z* for C_14_H_14_N_3_O_4_ ([M + H]^+^): calculated 288.0979, found 288.0978.

#### 2-(Aminomethyl)-5-((4-methoxybenzyl)oxy)-4*H*-pyran-4-one (6)

To a solution of 5 (2.33 g, 8.11 mmol) in methanol (20 mL) triphenylphosphine (3.19 g, 12,2 mmol) was added and the reaction mixture was stirred at rt for 1 h. Water (5 mL) was added and a formed precipitate was filtered off. Mother liquid was collected and methanol was removed *in vacuo*. To the residue 1 M HCl was added to pH 3 and the acidic water phase was extracted with ethyl acetate (2 × 100 mL). The pH of the water phase was adjusted to 12 using 1 M NaOH and alkaline water phase was extracted with ethyl acetate (2 × 100 mL). Combined organic phases were dried over Na_2_SO_4_, filtered and the solvent removed *in vacuo*. Yield: 0.786 g (37.1%); brown oil. ^1^H NMR (400 MHz, DMSO-*d*_6_) *δ* [ppm] 3.54 (s, 2H), 3.77 (s, 3H), 4.86 (s, 2H), 6.38 (s, 1H), 6.95 (d, *J* = 8.8 Hz, 2H), 7.35 (d, *J* = 8.8 Hz, 2H), 8.11 (s, 1H), signal for NH_2_ group is not seen. HRMS (ESI^+^) *m*/*z* for C_14_H_16_NO_4_ ([M + H]^+^): calculated 262.1074, found 262.1076.

#### 2-Amino-*N*-((5-((4-methoxybenzyl)oxy)-4-oxo-4*H*-pyran-2-yl)methyl)benzo[*d*]thiazole-6-carboxamide (8)

To the solution of 7 (362 mg, 1.86 mmol) in DMF (5 mL) EDC (535 mg, 2.23 mmol), HOBt (327 mg, 2.42 mmol) and *N*-methylmorpholine (409 μL, 3.72 mmol) were added and the reaction mixture was stirred on an ice bath for 20 min. 6 (487 mg, 1.86 mmol) was added and the reaction mixture was stirred at rt overnight. The precipitate in the reaction mixture was filtered off and dried to afford 8 as pale-yellow powder. Yield: 590 mg (72.4%); pale yellow powder; mp: 185–191 °C. ^1^H NMR (400 MHz, DMSO-*d*_6_) *δ* [ppm] 3.76 (s, 3H), 4.34 (d, *J* = 5.7 Hz, 2H), 4.85 (s, 2H), 6.26 (s, 1H), 6.95 (d, *J* = 6.8 Hz, 2H), 7.31–7.42 (m, 3H), 8.19 (s, 1H), 7.78 (dd, *J* = 1.9, 8.4 Hz, 1H), 7.82 (s, 2H), 8.22 (d, *J* = 1.8 Hz, 1H), 8.98 (t, *J* = 5.8 Hz, 1H). HRMS (ESI^+^) *m*/*z* for C_22_H_20_N_3_O_5_S ([M + H]^+^): calculated 438.1118, found 438.1119.

#### General procedure A. Synthesis of compounds 9 and 15a,b (with 9 as example)

To the solution of 8 (198 mg, 0.453 mmol) and 2,2,2-trichloro-1-(4,5-dibromo-1*H*-pyrrol-2-yl)ethan-1-one (167 mg, 0.453 mmol) in DMF (2 mL) Na_2_CO_3_ (48 mg, 0.453 mmol) was added and the reaction mixture was stirred at 80 °C overnight. Ethyl acetate (20 mL) and 1% citric acid (10 mL) were added and the precipitate that formed was filtered off and dried to give 9 (234 mg) as grey powder.

#### 2-(4,5-Dibromo-1*H*-pyrrole-2-carboxamido)-*N*-((5-((4-methoxybenzyl)oxy)-4-oxo-4*H*-pyran-2-yl)methyl)benzo[*d*]thiazole-6-carboxamide (9)

Yield: 234 mg (75.0%); grey powder; mp > 300 °C. ^1^H NMR (400 MHz, DMSO-*d*_6_) *δ* [ppm] 3.77 (s, 3H), 4.39 (d, *J* = 5.6 Hz, 2H), 4.86 (s, 2H), 6.31 (s, 1H), 6.95 (d, *J* = 8.7 Hz, 2H), 7.35 (d, *J* = 8.7 Hz, 2H), 7.55 (s, 1H), 7.84 (d, *J* = 8.4 Hz, 1H), 7.99 (dd, *J* = 1.8, 8.5 Hz, 1H), 8.20 (s, 1H), 8.55 (d, 1H, *J* = 1.8 Hz), 9.15 (t, 1H, *J* = 5.7 Hz), 12.82 (s, 1H), 13.28 (s, 1H). HRMS (ESI^+^) *m*/*z* for C_27_H_21_Br_2_N_4_O_6_S ([M + H]^+^): calculated 686.9543, found 686.9385.

#### General procedure B. Synthesis of compounds 10, 12 and 18a,b (with 10 as example)

To the solution of 9 (79 mg, 0.115 mmol) in acetic acid (3 mL) 1 M HCl in acetic acid (2.30 mL, 2.30 mmol) was added and the reaction mixture was stirred at rt overnight. The solvent was removed *in vacuo*, to the residue diethyl ether (10 mL) was added, sonicated and removed *in vacuo*. The procedure with diethyl ether was repeated 4 times. Finally, diethyl ether (10 mL) was added, sonicated and the resulting suspension was filtered to give the product (58 mg) as grey powder.

#### 2-(4,5-Dibromo-1*H*-pyrrole-2-carboxamido)-*N*-((5-hydroxy-4-oxo-4*H*-pyran-2-yl)methyl)benzo[*d*]thiazole-6-carboxamide (10)

Yield: 58 mg (88.9%); grey powder; mp > 300 °C. ^1^H NMR (400 MHz, DMSO-*d*_6_) *δ* [ppm] 4.38 (d, *J* = 5.5 Hz, 2H), 6.33 (s, 1H), 7.55 (d, *J* = 2.7 Hz, 1H), 7.83 (d, *J* = 8.5 Hz, 1H), 7.99 (dd, *J* = 1.8, 8.5 Hz, 1H), 8.08 (s, 1H), 8.57 (d, *J* = 1.7 Hz, 1H), 9.17 (s, 1H), 12.81 (s, 1H), 13.29 (d, *J* = 2.7 Hz, 1H), signal for NH group is missing. HRMS (ESI^+^) *m*/*z* for C_19_H_13_Br_2_N_4_O_5_S ([M + H]^+^): calculated 566.8968, found 566.8809. HPLC: *t*_r_ 5.38 min (81.5% at 254 nm).

#### General procedure C. Synthesis of compounds 11, 16a,b (with 11 as example)

To the suspension of 9 (100 mg, 0.145 mmol) in methanol (10 mL) 25% aq. NH_3_ solution (10 mL, 111 mmol) was added and the reaction mixture was stirred at 70 °C overnight. Methanol was removed *in vacuo*, the precipitate in the remaining mixture was filtered off and dried to give the product (88 mg) as light grey powder.

#### 2-(4,5-Dibromo-1*H*-pyrrole-2-carboxamido)-*N*-((5-((4-methoxybenzyl)oxy)-4-oxo-1,4-dihydropyridin-2-yl)methyl)benzo[*d*]thiazole-6-carboxamide (11)

Yield: 88 mg (87.9%); light grey powder; mp: 237–242 °C. ^1^H NMR (400 MHz, DMSO-*d*_6_) *δ* [ppm] 3.76 (s, 3H), 4.43 (s, 2H), 4.94 (s, 2H), 6.11 (s, 1H), 6.94 (d, *J* = 8.7 Hz, 2H), 7.29–7.45 (m, 3H), 7.54 (s, 1H), 7.84 (d, *J* = 8.5 Hz, 1H), 8.00 (dd, *J* = 1.8, 8.6 Hz, 1H), 8.56 (d, 1H, *J* = 1.8 Hz), 9.09 (t, 1H, *J* = 5.8 Hz), 11.22 (s, 1H), 12.82 (s, 1H), 13.28 (s, 1H). HRMS (ESI^+^) *m*/*z* for C_27_H_22_Br_2_N_5_O_5_S ([M + H]^+^): calculated 685.9703, found 685.9544.

#### 2-(4,5-Dibromo-1*H*-pyrrole-2-carboxamido)-*N*-((5-hydroxy-4-oxo-1,4-dihydropyridin-2-yl)methyl)benzo[*d*]thiazole-6-carboxamide (12)

Synthesized according to General procedure B using 2-(4,5-dibromo-1*H*-pyrrole-2-carboxamido)-*N*-((5-((4-methoxybenzyl)oxy)-4-oxo-1,4-dihydropyridin-2-yl)methyl)benzo[*d*]thiazole-6-carboxamide (11, 70 mg, 0.102 mmol) as reactant. Yield: 49 mg (84.8%); purple solid; mp > 300 °C. ^1^H NMR (400 MHz, DMSO-*d*_6_) *δ* [ppm] 4.65 (d, *J* = 5.6 Hz, 2H), 7.22 (s, 1H), 7.55 (s, 1H), 7.83 (d, *J* = 8.5 Hz, 1H), 7.99 (dd, *J* = 1.6, 8.5 Hz, 1H), 8.08 (s, 1H), 8.57 (d, *J* = 1.7 Hz, 1H), 9.41 (t, *J* = 5.7 Hz, 1H), 12.82 (s, 1H), 13.29 (d, *J* = 2.7 Hz, 1H), 14.36 (s, 1H). ^13^C NMR (101 MHz, DMSO-*d*_6_) *δ* [ppm] 99.63, 109.39, 110.83, 116.93, 117.22, 120.31, 122.10, 126.07, 126.16, 126.39, 129.12, 132.03, 144.68, 147.40, 151.41, 157.84, 161.25, 162.05, 166.98. HRMS (ESI^−^) *m*/*z* for C_14_H_13_Br_2_N_5_O_4_S ([M − H]^−^): calculated 565.9128, found 565.8969. HPLC: *t*_r_ 5.25 min (83.6% at 254 nm).

#### Methyl 2-amino-4-((5-((4-methoxybenzyl)oxy)-4-oxo-4*H*-pyran-2-yl)methoxy)benzo[*d*]thiazole-6-carboxylate (14)

To a solution of methyl 2-amino-4-hydroxybenzo[*d*]thiazole-6-carboxylate (1.02 g, 3.12 mmol) in acetonitrile (40 mL), K_2_CO_3_ (0.860 g, 6.22 mmol) and 2-(bromomethyl)-5-((4-methoxybenzyl)oxy)-4*H*-pyran-4-one (4, 0.700 g, 3.12 mmol) were added. Reaction was stirred under reflux overnight. Solvent was removed under reduced pressure and the crude residue dissolved in ethyl acetate (140 mL). Organic phase was then successively washed with 10% citric acid (180 mL) and saturated NaHCO_3_ solution (180 mL). The precipitated product was filtered off. Solvent was removed under reduced pressure and crude product purified by column chromatography using dichloromethane/methanol (20 : 1) as eluent. Yield: 0.89 g (60.5%); brown solid; mp: 126 °C. ^1^H NMR (400 MHz, DMSO-*d*_6_) *δ* [ppm] 3.76 (s, 3H), 3.83 (s, 3H), 4.88 (s, 2H), 5.17 (s, 2H), 6.57 (s, 1H), 6.96 (d, *J* = 8.7 Hz, 2H), 7.35 (d, *J* = 8.7 Hz, 2H), 7.44 (d, *J* = 1.5 Hz, 1H), 7.99 (s, 2H), 8.02 (d, *J* = 1.5 Hz, 1H), 8.27 (s, 1H).

#### Methyl 2-(4,5-dibromo-1*H*-pyrrole-2-carboxamido)-4-((5-((4-methoxybenzyl)oxy)-4-oxo-4*H*-pyran-2-yl)methoxy)benzo[*d*]thiazole-6-carboxylate (15a)

Synthesized according to General procedure A using methyl 2-amino-4-((5-((4-methoxybenzyl)oxy)-4-oxo-4*H*-pyran-2-yl)methoxy)benzo[*d*]thiazole-6-carboxylate (14, 350 mg, 0.75 mmol). Yield: 420 mg (77.8%); beige solid; mp: 254 °C. ^1^H NMR (400 MHz, DMSO-*d*_6_) *δ* [ppm] 3.78 (s, 3H), 3.89 (s, 3H), 4.90 (s, 2H), 5.26 (s, 2H), 6.64 (s, 1H), 6.97 (d, *J* = 8.7 Hz, 2H), 7.36 (d, *J* = 8.6 Hz, 2H), 7.57 (d, *J* = 2.7 Hz, 1H), 7.60 (d, 1H), 8.31 (d, 1H), 8.35 (s, 1H), 13.00 (s, 1H), 13.28 (s, 1H). MS (ESI) *m*/*z* = 643.8 ([M + H]^+^).

#### Methyl 2-(3,4-dichloro-5-methyl-1*H*-pyrrole-2-carboxamido)-4-((5-((4-methoxybenzyl)oxy)-4-oxo-4*H*-pyran-2-yl)methoxy)benzo[*d*]thiazole-6-carboxylate (15b)

Synthesized according to General procedure A using methyl 2-amino-4-((5-((4-methoxybenzyl)oxy)-4-oxo-4H-pyran-2-yl)methoxy)benzo[*d*]thiazole-6-carboxylate (14, 400 mg, 0.854 mmol) and 2,2,2-trichloro-1-(3,4-dichloro-5-methyl-1*H*-pyrrol-2-yl)ethan-1-one (166 mg, 0.854 mmol) as reactants. The precipitate after filtration contained a mixture of the starting compound (14) and the product. To separate, the mixture was suspended in methanol, heated and the precipitate which contained the starting compound was filtered off. The procedure was repeated three times to wash the product into methanol. Methanol was then left to cool at room temperature overnight, the product precipitated and was filtered off and dried. Yield: 135 mg (24.5%); light grey solid. ^1^H NMR (400 MHz, DMSO-*d*_6_) *δ* [ppm] 2.27 (s, 3H), 3.77 (s, 3H), 3.89 (s, 3H), 4.90 (s, 2H), 5.27 (s, 2H), 6.65 (s, 1H), 6.96 (d, *J* = 8.7 Hz, 2H), 7.36 (d, *J* = 8.6 Hz, 2H), 7.60 (s, 1H), 8.30 (s, 1H), 8.34 (s, 1H), 12.25 (s, 2H). HRMS (ESI^+^) *m*/*z* for C_29_H_24_Cl_2_N_3_O_8_S ([M + H]^+^): calculated 644.0656, found 644.0652.

#### Methyl 2-(4,5-dibromo-1*H*-pyrrole-2-carboxamido)-4-((5-((4-methoxybenzyl)oxy)-4-oxo-1,4-dihydropyridin-2-yl)methoxy)benzo[*d*]thiazole-6-carboxylate (16a)

Synthesized according to General procedure C using methyl 2-(4,5-dibromo-1*H*-pyrrole-2-carboxamido)-4-((5-((4-methoxybenzyl)oxy)-4-oxo-4*H*-pyran-2-yl)methoxy)benzo[*d*]thiazole-6-carboxylate (15a, 150 mg, 0.12 mmol). The crude product was purified with flash column chromatography using dichloromethane/methanol (9 : 1) as eluent. Compound was obtained as a mixture of tautomers and ^1^H NMR signals could not be unambiguously assigned. Yield: 48 mg (32.0%); beige solid; mp > 300 °C. MS (ESI) *m*/*z* = 717.3 ([M + H]^+^).

#### Methyl 2-(3,4-dichloro-5-methyl-1*H*-pyrrole-2-carboxamido)-4-((5-((4-methoxybenzyl)oxy)-4-oxo-1,4-dihydropyridin-2-yl)methoxy)benzo[*d*]thiazole-6-carboxylate (16b)

Synthesized according to General procedure C using methyl 2-(3,4-dichloro-5-methyl-1*H*-pyrrole-2-carboxamido)-4-((5-((4-methoxybenzyl)oxy)-4-oxo-4*H*-pyran-2-yl)methoxy)benzo[*d*]thiazole-6-carboxylate (15b, 279 mg, 0.432 mmol). The crude product was suspended in ethyl acetate and a drop of methanol, filtered off and dried to give 60 mg of the product as a brown solid. The mother liquid was additionally purified with flash column chromatography using dichloromethane/methanol (20 : 1 → 9 : 1) as eluent to give another 32 mg of the product as white crystals. Compound was obtained as a mixture of tautomers and ^1^H NMR signals could not be unambiguously assigned. Yield: 92 mg (33.0%); brown solid and white crystals. MS (ESI) *m*/*z* = 643.0 ([M + H]^+^).

#### 2-(4,5-Dibromo-1*H*-pyrrole-2-carboxamido)-4-((5-((4-methoxybenzyl)oxy)-4-oxo-1,4-dihydropyridin-2-yl)methoxy)benzo[*d*]thiazole-6-carboxylic acid (17a)

To a suspension of 16a (40 mg, 0.060 mmol) in 1,4-dioxane 2 M NaOH (140 μL, 0.280 mmol) was added and the reaction mixture was stirred at 60 °C overnight. Additional 140 μL of 2 M NaOH were added and the reaction mixture was stirred at 60 °C for another 3 h. The solvent was evaporated *in vacuo*, the residue was acidified with 1 M HCl and the precipitated product was filtered off and dried. Compound was obtained as a mixture of tautomers and ^1^H NMR signals could not be unambiguously assigned. Yield: 25 mg (63.7%); beige solid; mp > 300 °C. MS (ESI) *m*/*z* = 703.0 ([M − H]^−^).

#### 2-(3,4-Dichloro-5-methyl-1*H*-pyrrole-2-carboxamido)-4-((5-((4-methoxybenzyl)oxy)-4-oxo-1,4-dihydropyridin-2-yl)methoxy)benzo[*d*]thiazole-6-carboxylic acid (17b)

To a suspension of 16b (92 mg, 0.150 mmol) in methanol 1 M NaOH (750 μL, 0.750 mmol) was added and the reaction mixture was stirred at 60 °C overnight. Additional 750 μL of 1 M NaOH were added and the reaction mixture was stirred at 60 °C for another 3 h. The solvent was evaporated *in vacuo*, the residue was acidified with 1 M HCl and the precipitated product was filtered off and dried. Compound was obtained as a mixture of tautomers and ^1^H NMR signals could not be unambiguously assigned. Yield: 30 mg (33.3%); brown solid; mp > 300 °C. MS (ESI) *m*/*z* = 627.0 ([M − H]^−^).

#### 2-(4,5-Dibromo-1*H*-pyrrole-2-carboxamido)-4-((5-hydroxy-4-oxo-1,4-dihydropyridin-2-yl)methoxy)benzo[*d*]thiazole-6-carboxylic acid (18a)

Synthesized according to General procedure B using 2-(4,5-dibromo-1*H*-pyrrole-2-carboxamido)-4-((5-((4-methoxybenzyl)oxy)-4-oxo-1,4-dihydropyridin-2-yl)methoxy)benzo[*d*]thiazole-6-carboxylic acid (17a, 23 mg, 0.033 mmol) as reactant and stirring the reaction mixture for 48 h. Yield: 10 mg (52.4%), pink solid; mp > 300 °C. ^1^H NMR (400 MHz, DMSO-*d*_6_) *δ* [ppm] 5.38 (s, 2H), 7.13 (s, 1H), 7.55 (s, 1H), 7.59 (d, *J* = 1.3 Hz, 2H), 8.02 (s, 1H), 8.32 (d, *J* = 1.3 Hz, 1H), 12.94 (s, 1H), 13.29 (s, 1H), signals for NH and COOH groups are missing. HRMS (ESI^−^) *m*/*z* for C_19_H_11_Br_2_N_4_O_6_S ([M − H]^−^): calculated 580.8773, found 580.8772. HPLC: *t*_r_ 5.72 min (95.9% at 254 nm).

#### 2-(3,4-Dichloro-5-methyl-1*H*-pyrrole-2-carboxamido)-4-((5-hydroxy-4-oxo-1,4-dihydropyridin-2-yl)methoxy)benzo[*d*]thiazole-6-carboxylic acid (18b)

Synthesized according to General procedure B using 2-(3,4-dichloro-5-methyl-1*H*-pyrrole-2-carboxamido)-4-((5-((4-methoxybenzyl)oxy)-4-oxo-1,4-dihydropyridin-2-yl)methoxy)benzo[*d*]thiazole-6-carboxylic acid (17b, 27 mg, 0.043 mmol) as reactant. 60 equiv. of 1 M HCl in acetic acid were used and the reaction mixture was stirred in a pressure tube at 50 °C for 1 week. The crude product was suspended in methanol, filtered off and dried. Yield: 13.6 mg (62.3%); beige solid; mp > 300 °C. ^1^H NMR (400 MHz, DMSO-*d*_6_) *δ* [ppm] 2.27 (s, 3H), 5.50 (s, 2H), 7.43 (s, 1H), 7.61 (d, *J* = 1.4 Hz, 1H), 8.23 (s, 1H), 8.34 (d, *J* = 1.4 Hz, 1H), 11.12 (s, 1H), 12.12 (s, 1H), 12.41 (s, 1H), signals for NH and COOH groups are missing. HRMS (ESI^−^) *m*/*z* for C_20_H_13_Cl_2_N_4_O_6_S ([M − H]^−^): calculated 506.9938, found 506.9943. HPLC: *t*_r_ 5.11 min (90.0% at 254 nm).

### Determination of inhibitory activities against *E. coli* DNA gyrase and topoisomerase IV

The assays for determination of IC_50_ against *E. coli* DNA gyrase and topoisomerase IV were performed according to previously reported procedures.^23^ IC_50_ values were determined with seven concentrations of the inhibitors. GraphPad Prism program was used for calculating an IC_50_ value, which represents the concentration of inhibitor where the activity of the enzyme is reduced by 50%. IC_50_ values were determined in three independent measurements, and their average value is given as a final result.

### Determination of antibacterial activities

The clinical microbiology control strains of *E. coli* (ATCC 25922), *P. aeruginosa* (ATCC 27853) and *A. baumannii* (ATCC 19606) were obtained from Microbiologics Inc. (St. Cloud, MN, USA). The single-gene knock-out mutant strain of *E. coli* JW5503 (*tolC* knock-out) was obtained from the *E. coli* collection of the National BioResource Project at the National Institute of Genetics (Japan).^45^ To determine the antibacterial activities, broth microdilution assays were carried out in 96-well plates following the Clinical and Laboratory Standards Institute guidelines.^46^ Compounds were prepared as 100% DMSO stock solutions. The final concentration of DMSO was 1% and had no effect on the growth of bacteria. Preliminary screening was performed at 50 μM, and growth inhibition was measured after 24 h incubation at 37 °C. For compounds displaying >90% growth inhibition in the preliminary assay, MIC values were determined in dose–response assays (from at least two independent experiments, each with three replicates per concentration). Cation-adjusted Mueller-Hinton broth (CAMHB, BD) was used in all assays. CAMHB was prepared according to manufacturer's instructions and iron-depleted CAMHB (ID-CAMHB) according to CLSI guidelines. ID-CAMHB was used in order to mimic the conditions faced by bacteria during human infections, and induces the ferric iron transport. ID-CAMHB was prepared by adding 100 g of Chelex 100 resin (Bio-Rad) to 1 L of autoclaved CAMHB and stirred for 2 h at room temperature, in order to remove cations from the medium. Then, broth was filtered using a 0.2 μm filter to remove the resin and pH adjusted to 7.3 using 5 M hydrochloric acid. ID-CAMHB was then supplemented with calcium (CaCl_2_), magnesium (MgCl_2_) and zinc (ZnSO_4_) to final concentrations of 22.5 μg mL^−1^, 11.25 μg mL^−1^ and 0.56 μg mL^−1^, respectively, and filtered again. To evaluate the effect of iron concentration on antibacterial effect of compounds, an appropriate amount of iron(iii) chloride was added to ID-CAMHB, and this supplemented media was used as control CAMHB. The iron concentration in CAMHB and ID-CAMHB were approximately 0.2 mg L^−1^ and ≤0.03 mg L^−1^, respectively. Cefiderocol, a positive control, MICs were determined against *E. coli*, *P. aeruginosa* and *A. baumannii*, and used as positive control in the assays. Cefiderocol was purchased from MedChemExpress Europe.

#### Nuclear magnetic resonance

Spectra were recorded at 300 K on a Bruker Avance III HD 600 (Bruker, Billerica, MA, USA) operating at a ^1^H frequency of 600 MHz. The one-dimensional ^1^H spectra were acquired with a pulse of 10.0 μs (90°), 3 s recycle delay, 1.5 s acquisition time, and 16 transients over a spectral window of 9000 Hz. The titrations of 18b in DMSO-*d*_6_ were performed with FeCl_3_ in steps of 0.1 equivalents of Fe^3+^ up to 0.4, at constant ligand concentration in the 2–3 mM range.

#### Molecular docking

Molecular docking calculations were performed in Schrödinger Release 2022–1 (Schrödinger, LLC, New York, NY, USA, 2022). Crystal structures of *E. coli* GyrB (PDB entry: 4DUH) and ParE (PDB entry: 1S16) were retrieved from Protein Data Bank. Proteins were then prepared by Protein Preparation Wizard using default settings. Receptor grids were calculated for the ligand-binding sites and designed compounds were docked using Glide XP protocol as implemented in Schrödinger Release 2022-1 (Glide, Schrödinger, LLC, New York, NY, USA, 2022). The highest ranked docking pose was used for visualization.

#### Molecular dynamics simulations

MD simulation of 18b in complex with *E. coli* GyrB was performed using NAMD package (version 2.9)^47^ and the CHARMM36m^48^ force field. Removal of potential steric clashes and optimization of the atomic coordinates of the docking complex were first performed by steepest descent (10 000 steps) and adopted basis Newton–Raphson (10 000 steps) energy minimizations. The system for MD simulation was prepared using CHARMM-GUI.^49,50^ Molecular mechanics parameters for compound 18b were estimated using the ParamChem tool.^51–53^ Structure of the 18b–GyrB complex was first embedded in a box of TIP3P water molecules. Then the system was neutralized by addition of KCl. The MD simulation was run in the NPT ensemble using the periodic boundary conditions. Temperature (300 K) and pressure (1 atm) were controlled using the Langevin dynamics and Langevin piston methods, respectively. Short-range and long-range forces were calculated every 1 and 2 time steps, respectively, with a time step of 2.0 ps. The smooth particle mesh Ewald method was used to calculate the electrostatic interactions.^54^ The short-range interactions were cut off at 12 Å. All of the chemical bonds between hydrogen and the heavy atoms were held fixed using the SHAKE algorithm.^55^ After equilibration of the complete system for 1 ns, an unconstrained 100 ns production was run.

#### Structure-based pharmacophore modeling

The 100 ns MD trajectory of *E. coli* GyrB in complex with 18b was used for pharmacophore feature analysis using LigandScout 4.4 Expert,^56^ which resulted in 1000 structure-based pharmacophore models.

## Conflicts of interest

There are no conflicts to declare.

## Supplementary Material

RA-014-D3RA08337C-s001
