# Supplementary material for: Benzothiazole DNA gyrase inhibitors and their conjugates with siderophore mimics: design, synthesis and evaluation
Source: RSC Adv. 2024 Jan 18;14(5):2905–17. doi: 10.1039/d3ra08337c (PMC10794952; doi:10.1039/d3ra08337c)

Electronic Supplementary Material (ESI) for RSC Advances.
This journal is © The Royal Society of Chemistry 2024

SUPPORTING INFORMATION

# Benzothiazole DNA gyrase inhibitors and their conjugates with siderophore mimics: Design, synthesis and evaluation

Martina Durcik,[a] Cristina D. Cruz,[b] Mariano Andrea Scorciapino,[c] Janez Ilaš,[a] Päivi Tammela,[b] Matteo Ceccarelli,[d] Lucija Peterlin Mašič[a] and Tihomir Tomašič,[a,*]

[a]*University of Ljubljana, Faculty of Pharmacy, Aškerčeva cesta 7, 1000 Ljubljana, Slovenia*

[b]*Drug Research Program, Division of Pharmaceutical Biosciences, Faculty of Pharmacy, University of Helsinki, P.O. Box 56 (Viikinkaari 5 E), FI-00014 Helsinki, Finland*

[c]*Department of Chemical and Geological Sciences, University of Cagliari, Cittadella Universitaria di Monserrato – S.P. 8 km 0.700, 09042 - Monserrato (CA), Italy*

[d]*Department of Physics and IOM/CNR, Sezione di Cagliari, University of Cagliari, Cittadella Universitaria di Monserrato – S.P. 8 km 0700, 09042 - Monserrato (CA), Italy*

***Corresponding author: Tihomir Tomašič**

University of Ljubljana, Faculty of Pharmacy,

Aškerčeva cesta 7, 1000 Ljubljana, Slovenia

Tel: +386-1-4769556

E-mail: tihomir.tomasic@ffa.uni-lj.si

**Representative analytical data (NMR spectra, HRMS spectra, HPLC chromatograms)**

$^{1}$H NMR spectrum of 2-(4,5-dibromo-1*H*-pyrrole-2-carboxamido)-*N*-((5-hydroxy-4-oxo-4*H*-pyran-2-yl)methyl)benzo[*d*]thiazole-6-carboxamide (**10**).

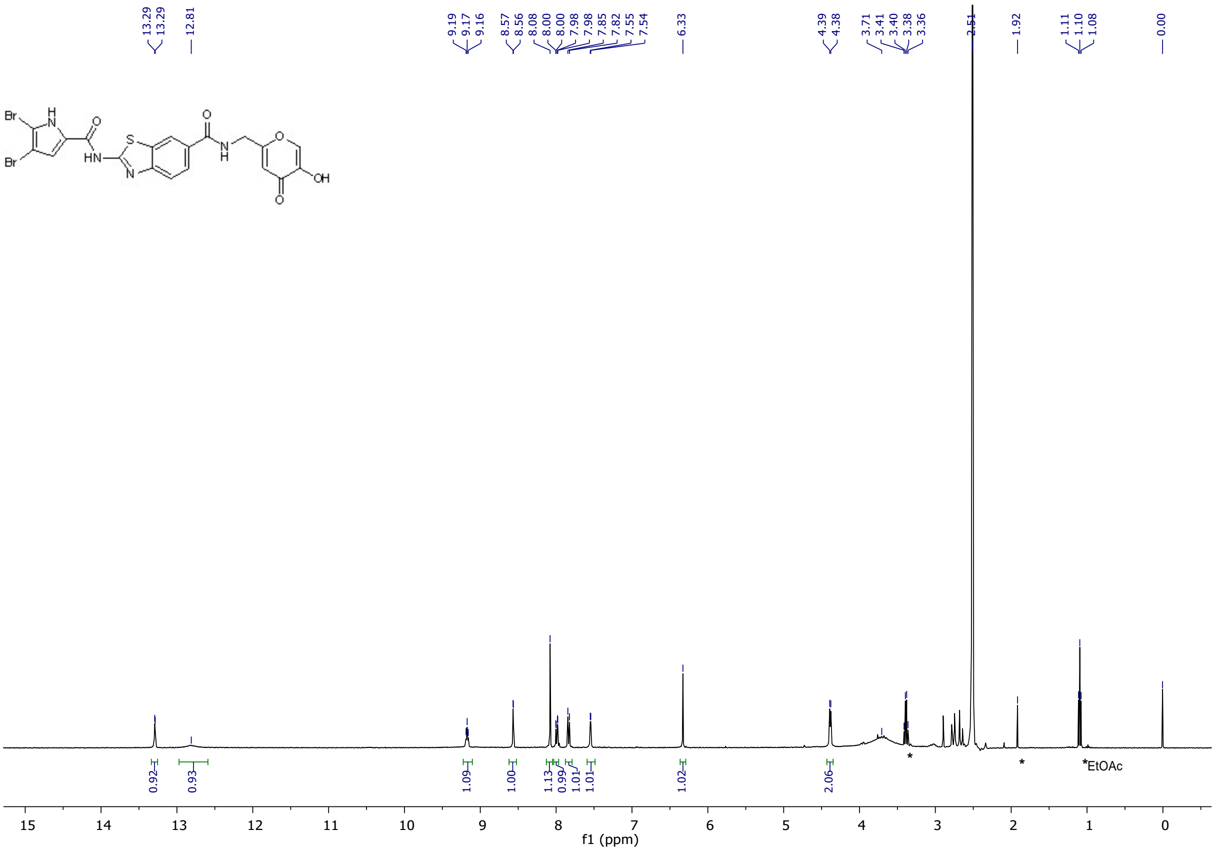

$^{1}$H and $^{13}$C NMR spectrum of 2-(4,5-dibromo-1*H*-pyrrole-2-carboxamido)-*N*-((5-hydroxy-4-oxo-1,4-dihydropyridin-2-yl)methyl)benzo[*d*]thiazole-6-carboxamide (**12**).

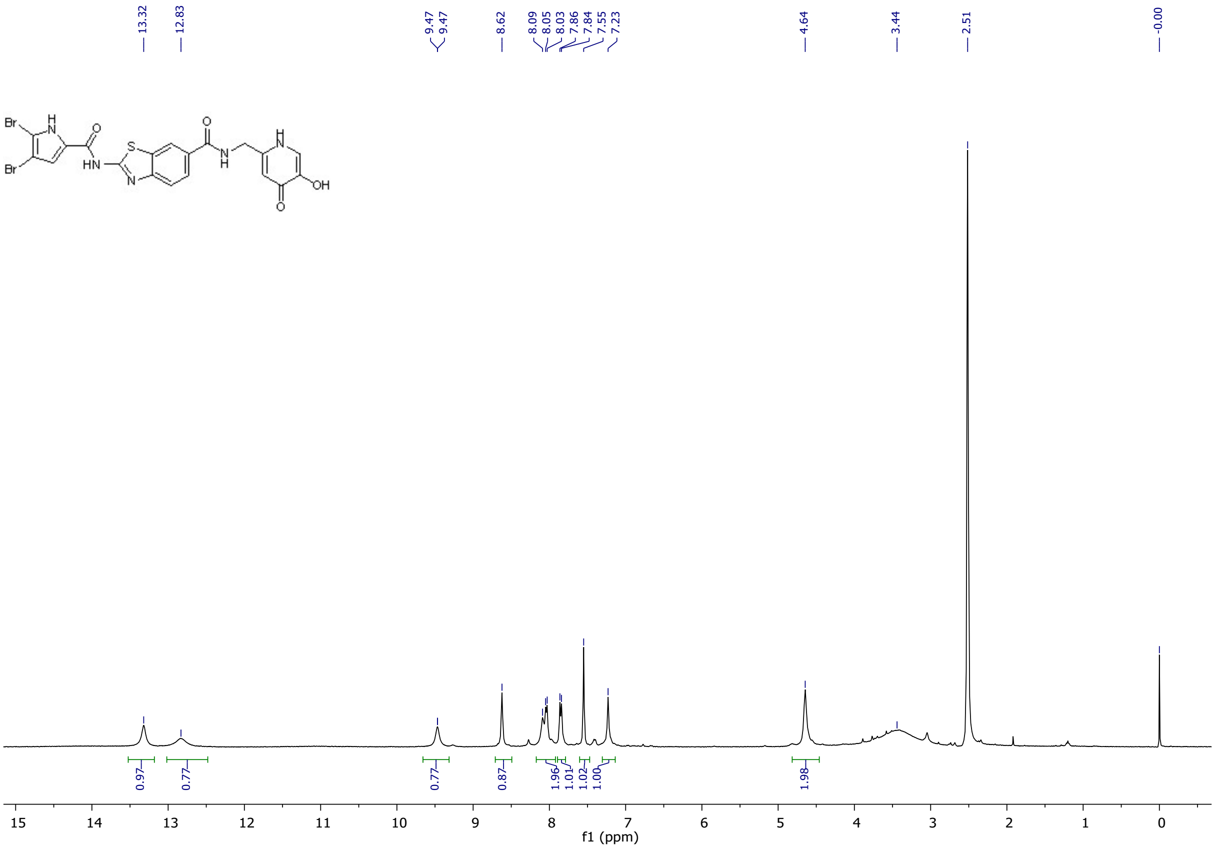


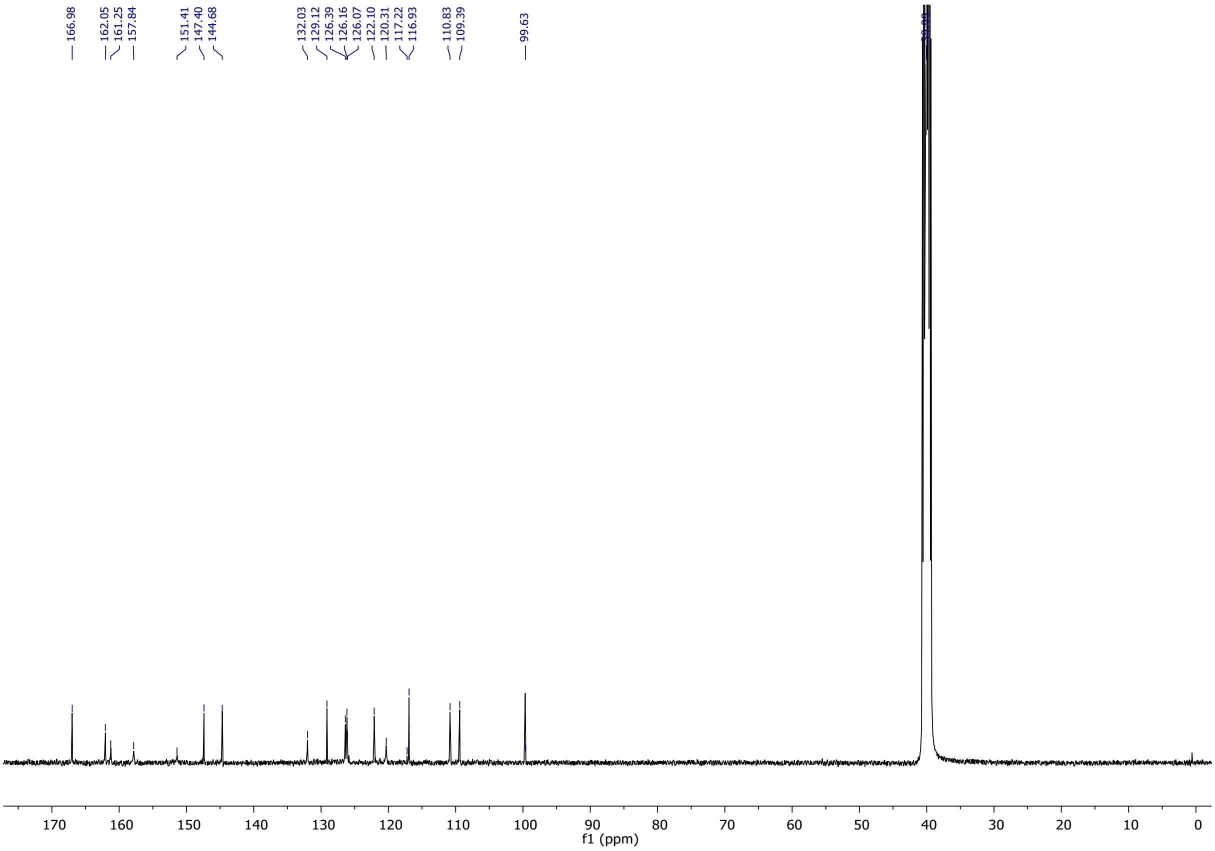

$^1$H NMR spectrum of 2-(4,5-dibromo-1*H*-pyrrole-2-carboxamido)-4-((5-hydroxy-4-oxo-1,4-dihydropyridin-2-yl)methoxy)benzo[*d*]thiazole-6-carboxylic acid (**18a**).

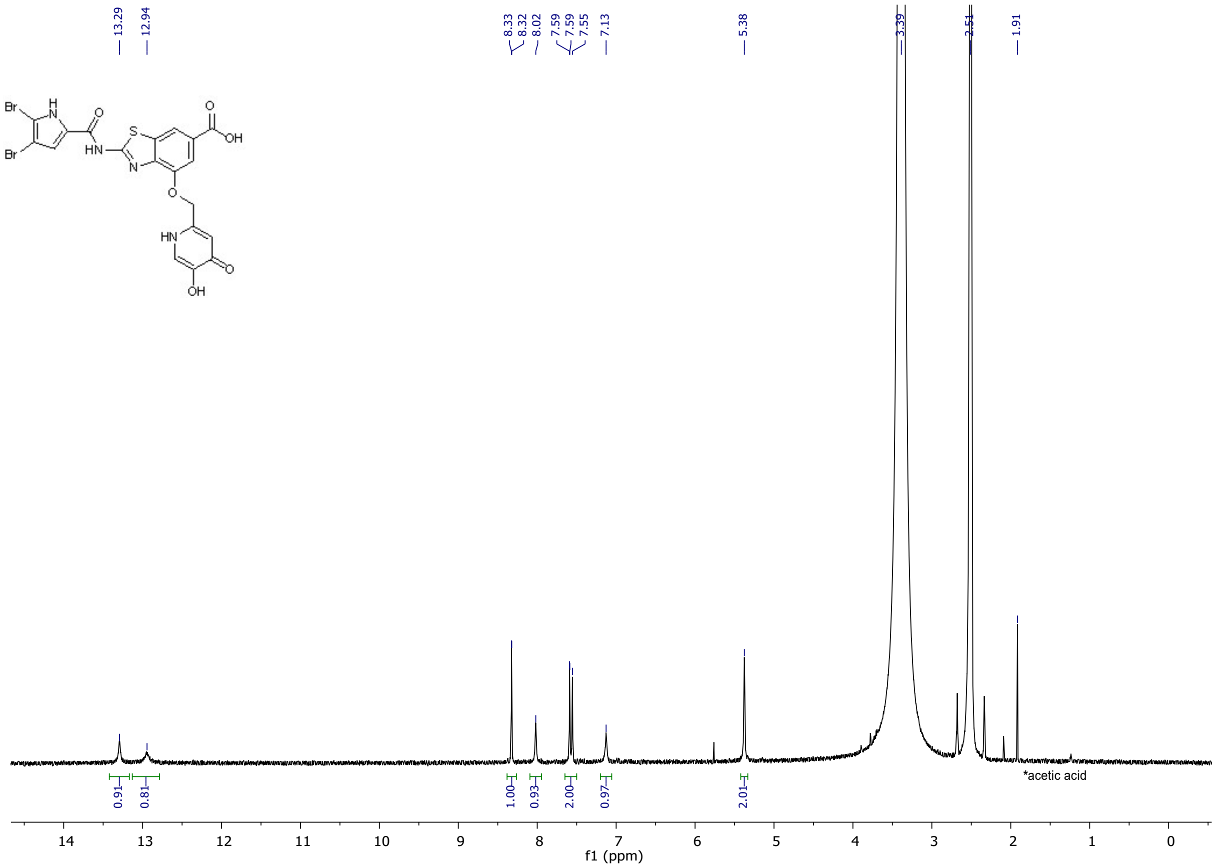

$^{1}$H NMR spectrum of 2-(3,4-dichloro-5-methyl-1*H*-pyrrole-2-carboxamido)-4-((5-hydroxy-4-oxo-1,4-dihydropyridin-2-yl)methoxy)benzo[*d*]thiazole-6-carboxylic acid (**18b**).

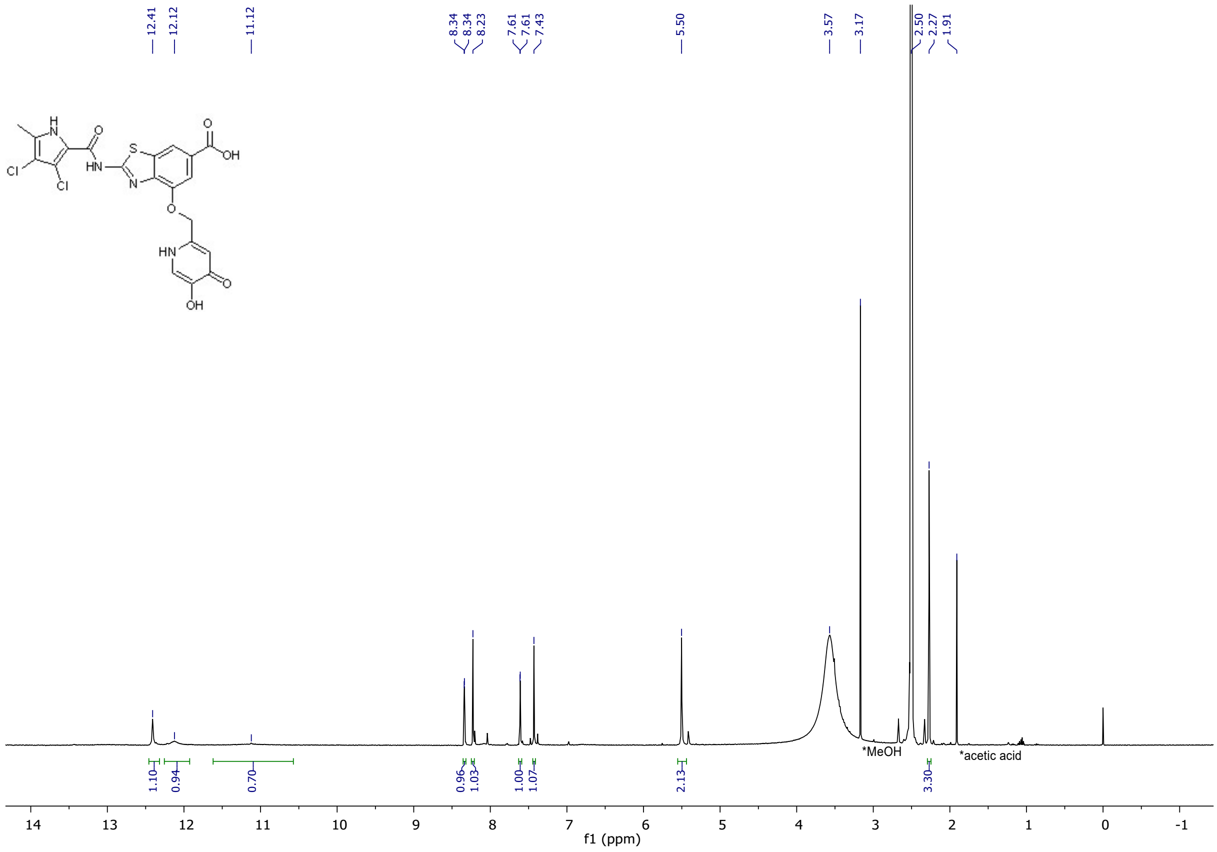

HRMS data for compound **18a**.

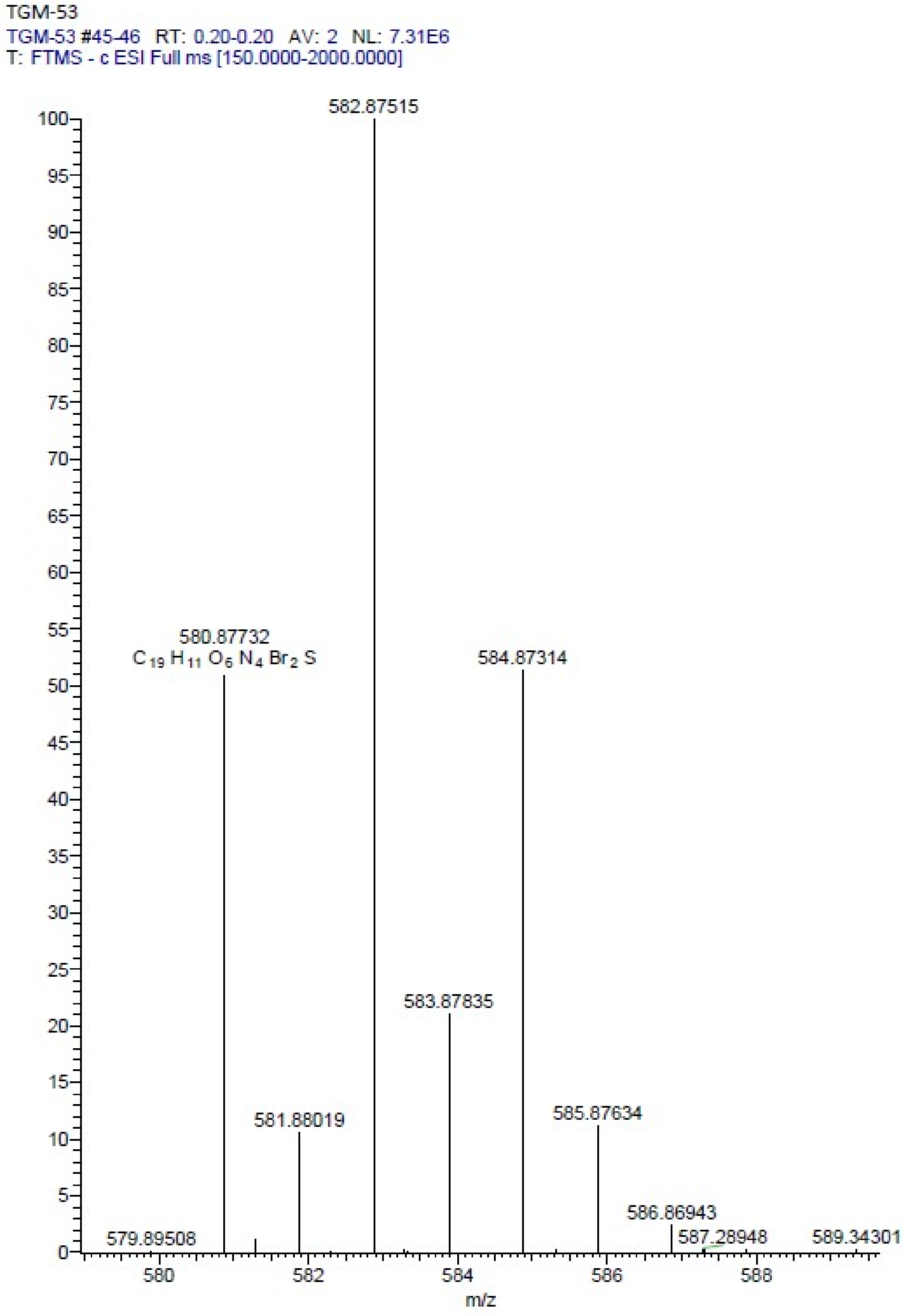


Elemental composition search on mass 580.87732

m/z= 575.87732-585.87732

| m/z | Theo. Mass | Delta (ppm) | RDB equiv. | Composition |
|---|---|---|---|---|
| 580.87732 | 580.87715 | 0.29 | 15.5 | $C_{19}H_{11}O_6N_4Br_2S$ |

HPLC traces for compound **18a**.

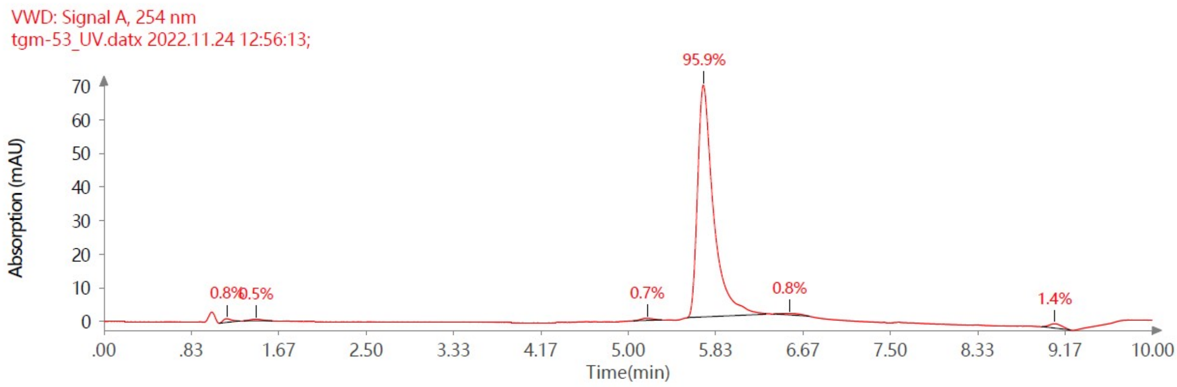


HPLC traces for compound **18b**.

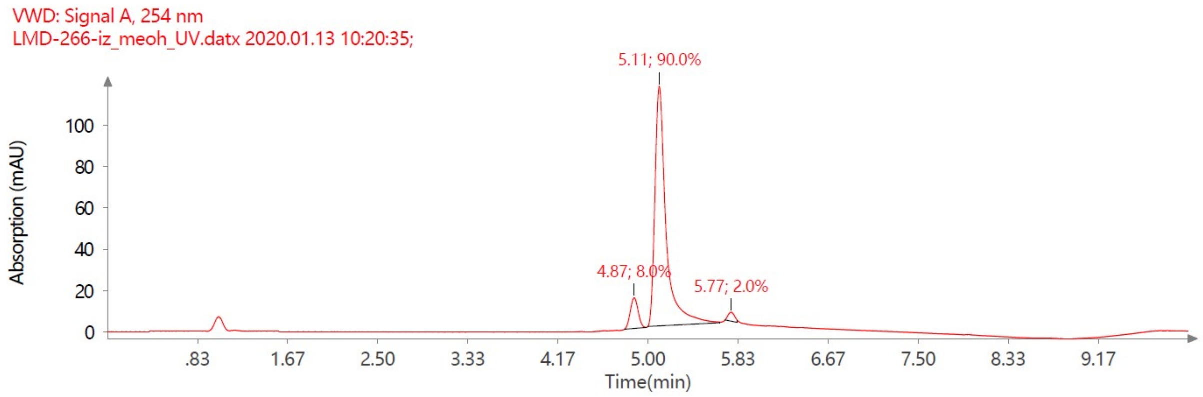

Supplement: RA-014-D3RA08337C-s001 [file RA-014-D3RA08337C-s001.pdf]
